# Subcutaneous lipomas: A minimally invasive method for resection of subcutaneous lipomas preserving retaining ligaments

**DOI:** 10.1007/s00238-017-1328-5

**Published:** 2017-06-09

**Authors:** Akio Sakamoto, Takeshi Okamoto, Shuichi Matsuda

**Affiliations:** Department of Orthopaedic Surgery, Graduate School of Medicine, Kyoto University, Shogoin, Kawahara-cho 54, Sakyo-ku, Kyoto, 606-8507 Japan

**Keywords:** Lipoma, Subcutis, Retaining ligament, Resection, Technique

## Abstract

**Background:**

Lipomas are common benign tumors usually located in the subcutaneous tissues. Resection of lipomas frequently requires incisions equal to the diameter of the tumor. The “squeeze technique” with a small incision is well-described, but is frequently not successful, particularly for lipomas in the shoulder region. We report a method for resection of subcutaneous lipomas that preserves retaining ligaments.

**Methods:**

Lipomas are characterized by high signal intensity on T1- and T2-weighted images on magnetic resonance imaging. Retaining ligaments demonstrate low signal intensity on T1-weighted images and high signal intensity on T2-weighted images with fat-suppression. Through a 1 in. incision, lipomas were detached from the retaining ligaments bluntly with a finger. Tumors were then extracted either in a piecemeal fashion or with the “squeeze technique.” Complete lipoma resection was visually confirmed through the incisions. For the current report, we analyzed 18 large lipomas resected by this method, with “large” defined as equal to or greater than 5 cm in diameter.

**Results:**

The 18 patients included four males and 14 females with a mean age of 53.4 (26–72). The mean lipoma size was 6.6 cm (5–12). Locations included the shoulder in nine cases (50%), the upper arm in five cases (28%), the back in two cases (11%), and the thigh in two cases (11%). Retaining ligaments were identified by MRI in all cases. Lipomas were located between retaining ligaments at the periphery of the tumor. All three lipomas larger than 10 cm were located in the shoulder. There was no difference in the technical difficulty of resection of these compared with lipomas less than 10 cm in diameter. There were no cases of chronic pain or residual hypoesthesia at the incision sites.

**Conclusions:**

The method is an easy and minimally invasive way to achieve complete resection, even for large lipomas, regardless of anatomical location. The method may contribute to reduction of side effects including residual hypoesthesia and chronic pain at the incision site, due to the small incision and preservation of retaining ligaments, which may contain cutaneous nerves.

Level of Evidence: Level IV, therapeutic study.

## Introduction

Lipomas are benign, slow-growing tumors that are usually located in the subcutaneous tissues. Surgical excision is the mainstay of treatment for lipomas [1], though they frequently require an incision equal to the diameter of the tumor. Small incisions are cosmetically beneficial [2], and possibly decrease pain and/or hypoesthesia at the incision. The “squeeze technique” with a small incision over the lipoma is a well-described technique for forearm or leg lipomas, but is frequently not successful for large lipomas, particularly those at the shoulder [3, 4]. Liposuction has also been reported as a potential minimally invasive treatment. However, long-term results of liposuction are disappointing in terms of completeness of resection and frequency of side-effects, especially when the lipoma is fibrous [2, 5].

Retaining ligaments are vertical condensations of the fascia that tether the skin to the deep fascia, possibly contributing to mechanical stabilization of the skin layers [6, 7]. Failure of the “squeeze technique” is believed to be due to the existence of retaining ligaments. In the present report, emphasis is put on retaining ligaments at the periphery of the lipomas. In our series, lipomas, including some in the shoulder region, were treated through a small incision with blunt tumor detachment.

## Methods

**Fig. 1:**
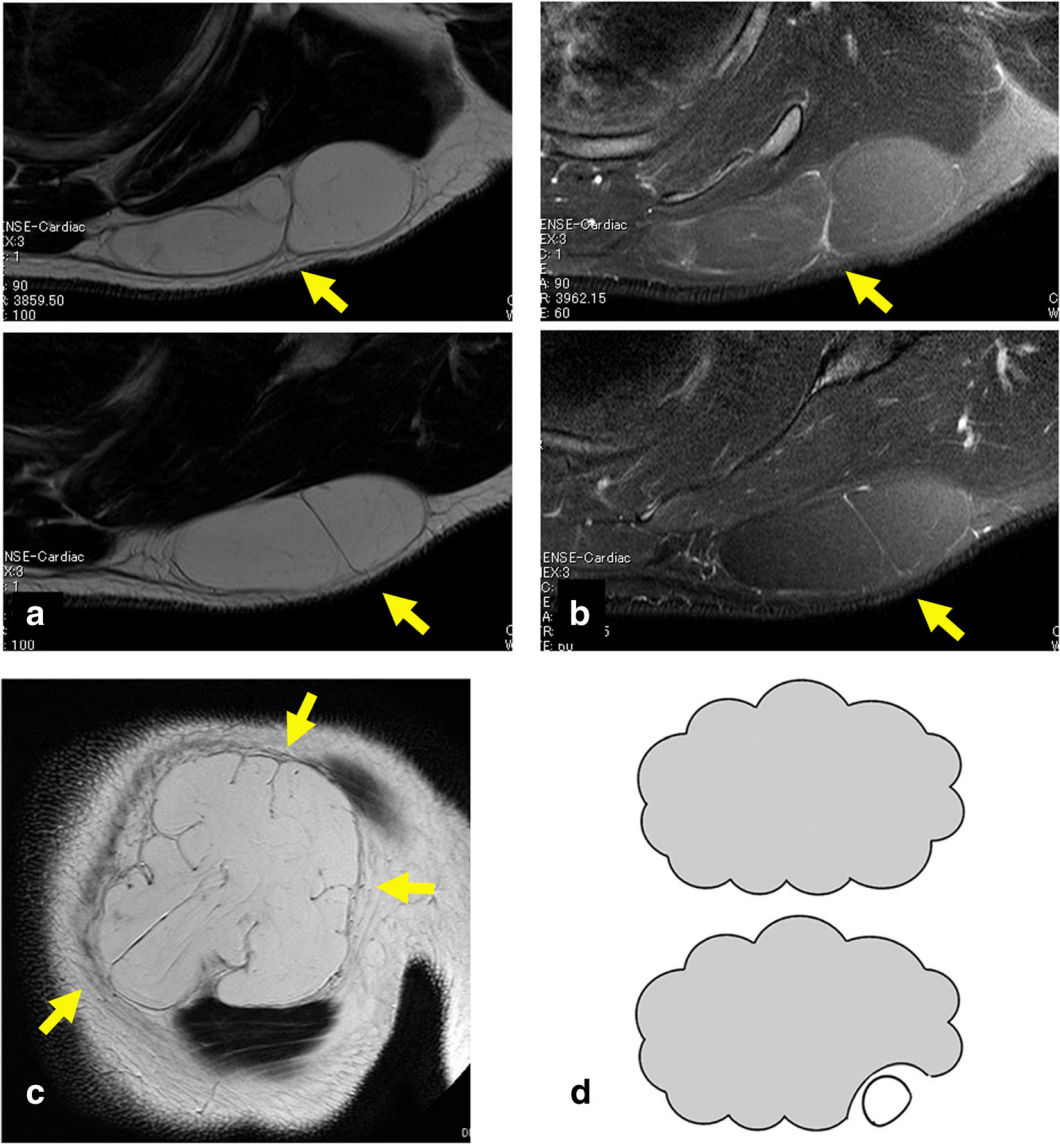
A 37-year-old man with a shoulder lipoma. The lipoma is characterized by high-signal intensity on T1-weighted images. Retaining ligaments (indicated by *yellow arrows*) are seen to have low-signal intensity on T1-weighted images (**a**). Suppressed signals on T2-weighted images with fat-suppression suggest a lipomatous tumor. The retaining ligaments show a high signal intensity (*yellow arrows*) (**b**). *Top* and *bottom* images are sequential (**a**, **b**). Coronal, T1-weighted images show the lipoma with notch formations representing retaining ligaments at the periphery (**c**). Resection schemas for blunt dissection are shown in *yellow arrows*. Using this method, the lipoma is detached bluntly with a finger; before (*top*) and after (*bottom*) the procedure (**d**)

The initial diagnosis of lipoma was made by magnetic resonance imaging (MRI) with high signal intensity on T1- and T2-weighted images; the signal pattern was similar to that of subcutaneous adipose tissue. The high-signal intensity was suppressed on T2-weighted images with fat-suppression (Fig. 1a). Retaining ligaments are located between the fascia and the skin. They demonstrate low signal intensity on T1-weighted images and high signal intensity on T2-weighted images with fat-suppression (Fig. 1b, c).

As for resection method, with an incision measuring 1 in, detachment of the lipoma from the retaining ligaments was performed bluntly with a finger, pulling the tumor between the retaining ligaments (Fig. 1d). The released lipoma was extracted in a piecemeal fashion, or via the “squeeze technique,” through the incision. Detachment and extraction were repeated until complete resection. Complete resection was confirmed visually through the incision.

In order to evaluate the usefulness of this method, we chose to include large lipomas, defined as having a diameter greater than or equal to 5 cm. We assessed clinical data, side-effects, and detectability of retaining ligaments using MRI. Variance in subject age and tumor size by location was analyzed with the Mann*-*Whitney *U* test. A *p* value less than 0.05 was considered statistically significant.

## Results

Eighteen cases of large, subcutaneous lipoma resection were included in our analysis. Diagnosis of lipoma was confirmed histopathologically in all cases. Clinical data is shown in Table 1. All lipomas were resected under general anesthesia. The patients included four males and 14 females, with a mean age of 53.4 ± 12.0 (range 26 to 72). Tumor locations included the shoulder in nine cases (50%), the upper arm in five cases (28%), the back (11%) in two cases, and the thigh in two cases (11%). There was no significant difference in age based on location. Mean size was 6.6 ± 2.1 cm (range 5 to 12 cm). Three cases of lipoma larger than 10 cm were all located in the shoulder.

**Table 1:** Clinical summary of 18 cases with large subcutaneous lipomas

Location	Case *N* (%)	M:F	Mean age, *y*	R:L	Size	Retaining ligaments^a^
Shoulder	9 (50%)	2:7	49.6 (38–60)	3:6	7.1 (5–12)	9/9 (100%)
Upper arm	5 (28%)	1:4	63.0 (48–72)	2:3	6.2 (5–7)	5/5 (100%)
Back	2 (11%)	0:2	42.0 (26–58)	1:1	6.0 (5–7)	2/2 (100%)
Thigh	2 (11%)	1:1	58.0 (54–62)	1:1	5.5 (5–6)	2/2 (100%)
Total lesions	18	4:14 (1:3.5)	53.4 ± 12.0 (26–72)	7:11 (1:1.6)	6.6 ± 2.1 (5–12)	18/18 (100%)

*F* female, *L* left, *M* male*, N* number*, R* right, *y* year

^a^Detected in magnetic resonance imaging

Retaining ligaments were seen on MRI surrounding the lipoma in all 18 cases (Fig. 1). Complete resection was easily confirmed visually through the incision given postoperative skin laxity. There was no difference in the technical difficulty of resection of these compared with lipomas less than 10 cm in diameter. No patients complained of chronic pain or residual hypoesthesia at the incision sites postoperatively.

## Discussion

The etiology for most lipomas is idiopathic. However, multiple lipomas are associated with hereditary multiple lipomatosis or Gardner syndrome [8]. Other lipomatous conditions include Dercum’s disease, characterized by multiple painful subcutaneous lipomas [9] and Madelung disease, characterized by benign symmetric lipomatosis of the head, neck, shoulders, and proximal upper extremities in men with heavy alcohol consumption [10]. An association with trauma has also been postulated, but the discovery of the lipomas in this condition is incidental [11, 12].

**Fig. 2:**
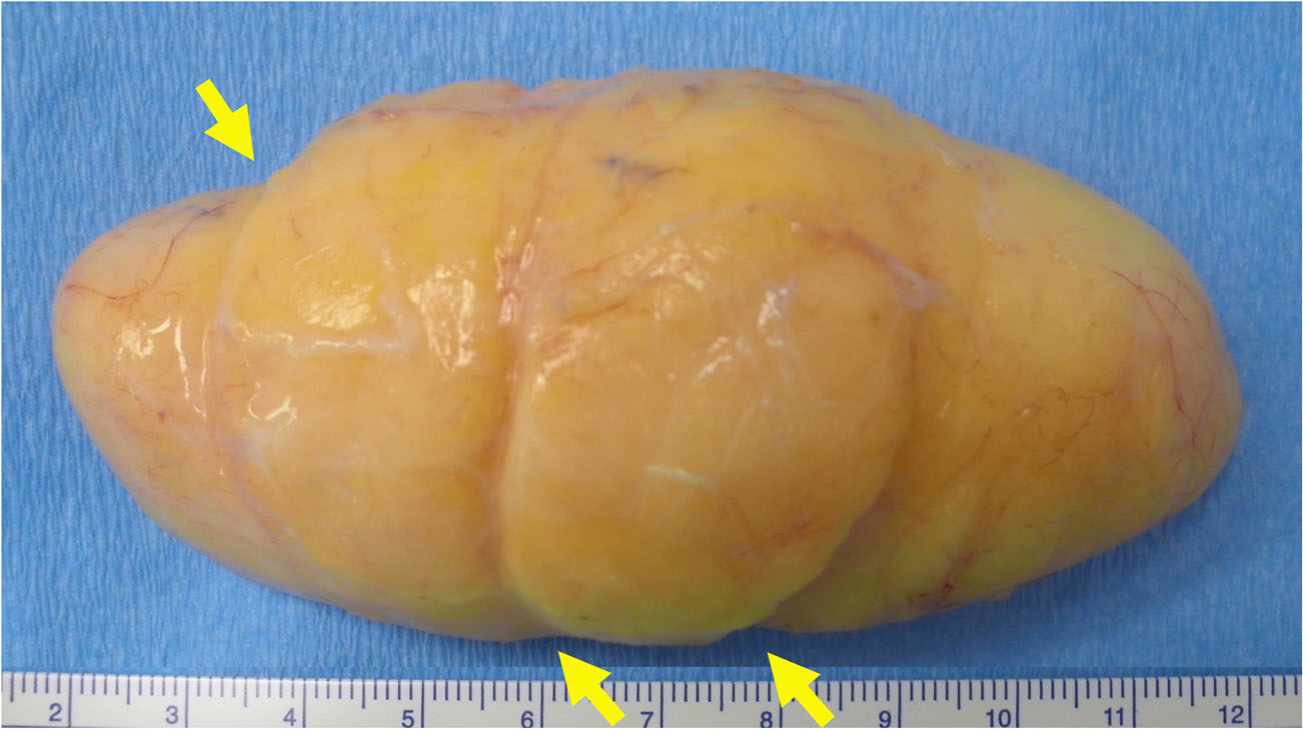
A 75-year-old woman with a lipoma (not included in the series). Notch formations indicated by *yellow arrows* representing retaining ligaments are seen on the side of the lipoma

In the present report, we focused on identification of retaining ligaments around lipomas (Fig. 2). The diagnosis of subcutaneous lipoma can be made on MRI, which demonstrates a signal pattern identical to subcutaneous adipose tissue. The lobular appearance seen in lipoma is also reported in other benign lipomatous tumors, including lipoblastoma, chondroid lipoma, and spindle cell lipoma [13]. The existence of retaining ligaments may be related to the lobulous appearance of benign lipomatous tumors occurring in the subcutaneous tissue.

The retaining ligaments are depicted as linear structures on MRI between the fascia to the skin. The ligaments are not truly linear, but rather membranous when taken as a series of MRI images. The presence of retaining ligaments was observed in all cases. Furthermore, the retaining ligaments were located at the periphery of the lipoma, and were continuous with the surrounding normal tissue. Retaining ligaments are characterized by high signal intensity on T2-weighted images with fat-suppression, suggesting that the retaining ligaments contain non-fibrous components, such as vessels or nerves. In a previous report about retaining ligaments in the face, the skin retaining ligaments are identified as serving a sentinel role with regard to peripheral nerve branches [14, 15]. The retaining ligaments might contain cutaneous nerves. In support of this, no patient in our series complained of hypoesthesia or chronic pain at the incision site, though the possibility that the small incision reduced the incidence of this side effect cannot be excluded.

The “squeeze technique” describes lipoma resection through a small incision overlying the lesion [3, 4]. This technique is performed by many surgeons. However, the success rate of the “squeeze technique” varies depending on the anatomical location. In a previous report on the “squeeze technique,” there was successful resection of 41 forearm lipomas, but not of 11 shoulder lipomas [4]. In the current series, all shoulder lipomas were successfully resected, and there seemed to be no difference in technical difficulty compared to lipomas at other sites. The reason that the “squeeze technique” is thought to not be successful is because of the presence of fibrous stroma, which are described as retaining ligaments in the current series. Detachment of the lipoma from the retaining ligaments with a finger allowed for extraction in a piecemeal fashion or via the “squeeze technique” through a small incision. Furthermore, the 1 in. incision was sufficient to confirm complete resection given skin laxity. The current technique was successfully applied to lipomas larger than 10 cm located at the shoulder.

In the current article, subcutaneous, large lipomas were resected through small incisions, accompanied by blunt finger detachment, with emphasis placed on preservation of retaining ligaments at the periphery of the lipomas. This method is an easy and minimally invasive way to achieve complete resection, even for large lipomas, regardless of anatomical location. This method may contribute to reduction of risks of excision, including residual hypoesthesia and chronic pain at the incision site. This reduction may be due to the small incision and preservation of retaining ligaments, which may contain cutaneous nerves. In order to confirm involvement of nerves in the retaining ligaments, further prospective comparative studies and histological investigation are required.
